# Hepatitis B virus infection after immunization: How serious it is? An updated review

**DOI:** 10.1007/s10238-025-01645-8

**Published:** 2025-04-10

**Authors:** Arezoo Marjani, Seyed Moayed Alavian, Mohssen Nassiri Toosi, Seyed Hoda Alavian, Mohammad Foad Abazari, Azam Khamseh, Seyed Mohammad Jazayeri

**Affiliations:** 1 Department of Virology, School of Public Health, Tehran University of Medical Sciences, Tehran, Iran; 2 Research Center for Clinical Virology, Tehran University of Medical Sciences, Tehran, Iran; 3 Middle East Liver Disease Center, Tehran, Iran; 4 Liver Transplantation Research Center, Tehran University of Medical Sciences, Tehran, Iran; 5 Middle East Liver Disease Center, Tehran, Iran; 6 Division of Medical Sciences, Island Medical Program, University of British Columbia, Victoria, BC Canada; 7 Department of Bacteriology and Virology, School of Medicine, Shiraz University of Medical Science, Shiraz, Iran

**Keywords:** Hepatitis B virus, Hepatocellular carcinoma, Vaccination, Hepatitis B third-generation vaccines

## Abstract

Infection with hepatitis B virus (HBV) is one of the significant challenges worldwide. Despite the availability of antiviral drugs against this virus, the most critical strategy to prevent HBV infection is HB vaccination. Basically, despite widespread conventional HB vaccination, due to various reasons, including waning of hepatitis B surface antibody (HBsAb) titer after vaccination, the emergence of vaccine-escape mutants, failure to respond to the vaccine due to viral and host factors, levels of response in high-risk individuals and non-responders to conventional HB vaccination remains a major, unsolved and severe concern. This review focuses on the underlying reasons for conventional hepatitis B vaccination failures. It also suggests solutions to overcome these failures by highlighting significant advances in vaccination, including hepatitis B third-generation vaccines and adjuvanted hepatitis B vaccines as efficient alternatives to second-generation vaccines. Potentially, these new strategies will compensate for the shortcomings caused by second-generation vaccines. Adherence to these denouements has a significant role in preventing the circulation of HBV among individuals and reducing the global burden of HBV-related diseases.

## Introduction

Infection with hepatitis B virus (HBV) remains a significant public health issue worldwide. According to the latest reports published in 2019, approximately 296 million individuals are struggling with chronic hepatitis B infection, with about 1.5 million new cases diagnosed annually [1–3]. Subsequently, chronic and acute infection caused by this virus causes severe damage to the liver, which following chronic infection for a very long time and gradually causes irreversible consequences such as cirrhosis and hepatocellular carcinoma (HCC) [2, 4]. Despite the availability of widespread antiviral treatments, these antiviral drugs are not able to perfectly clear virus inside the nucleus; hence, the best method to control the infection is through vaccination [5]. However, still, HBV infection following vaccination has become a considerable public health concern. Moreover, the lack of antibodies in immunocompromised individuals and the emergence of vaccine-escape mutants lead to inadequate response to vaccination. The present review focuses on the complicated problems following effective HBV vaccination, highlighting the reasons for the emergence of infection. Also, strategies such as the use of booster doses, third-generation HB vaccines and new adjuvanted vaccines are suggested as either the best alternative to conventional HB vaccines or as additives to previous vaccinations (regarding boosters) (Table 1).

**Table 1:** Development of hepatitis B vaccines to date

Types of HBV vaccines	Year	Generation	Preparation process
Plasma-derived HBV vaccines	1981	First-generation HBV vaccine	Purification of HBsAg in plasma obtained from HBsAg carriers
HBV DNA recombinant vaccine	1986	Second-generation HBV vaccines	Expression of the HBsAg in yeast cells
Expression of pre-S/S2 in mammalian cells	1990	Third-generation HBV vaccines	Pre-S/S2 or S, pre-S1, pre-S2 expression in mouse/CHO cell lines

*CHO* Chinese hamster ovary

## A brief overview of hepatitis B vaccines

### Plasma-derived vaccines

**Fig. 1:**
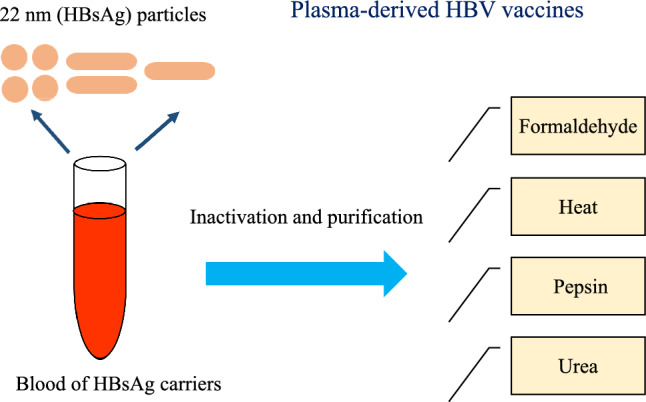
Production steps of the first generation of HB vaccines

Following the discovery of the Australian antigen and the observation of Dane particles using an electron microscope, progress in the production of the hepatitis B vaccine has been significant [6, 7]. Since there is no successful in vitro cell culture for HBV replication, it seems unlikely to produce hepatitis B vaccine by cell culture techniques. The development of the hepatitis B vaccine was based on Dr Krugman’s efforts and findings. In this line, significant progress was made in the production of hepatitis B vaccine by eliminating the infectivity of this virus by boiling the plasma of HBV carriers. After receiving the boiled plasma from infected individuals, the production of anti-HBs antibodies against HBsAg was induced, followed by procedures which resulted in partial protection against the virus [8, 9]. In the HBV carriers, HBV antigens are produced entirely naturally, which provides the prospect of making a hepatitis B vaccine [7]. Considering the susceptibility of chimpanzees to human HBV infection, it was possible to introduce a suitable animal model to assess the safety and efficacy of hepatitis B vaccine [10]. HBV challenge showed the protection of vaccinated chimpanzees by the purified HBsAg [11]. Therefore, in the early 1980s, in countries such as the USA and France, using 22 nm hepatitis B surface antigen (HBsAg) collected and obtained from HBsAg carriers, so-called plasma-derived vaccines were made as the first-generation vaccines against hepatitis B. Purification and inactivation of the first-generation vaccines was done through treatments with heat, formaldehyde, pepsin and urea. Following vaccination with plasma-derived vaccines, millions of individuals have proven satisfactory protection with adequate effectiveness and safety [12, 13] (Fig. 1).

**Fig. 2:**
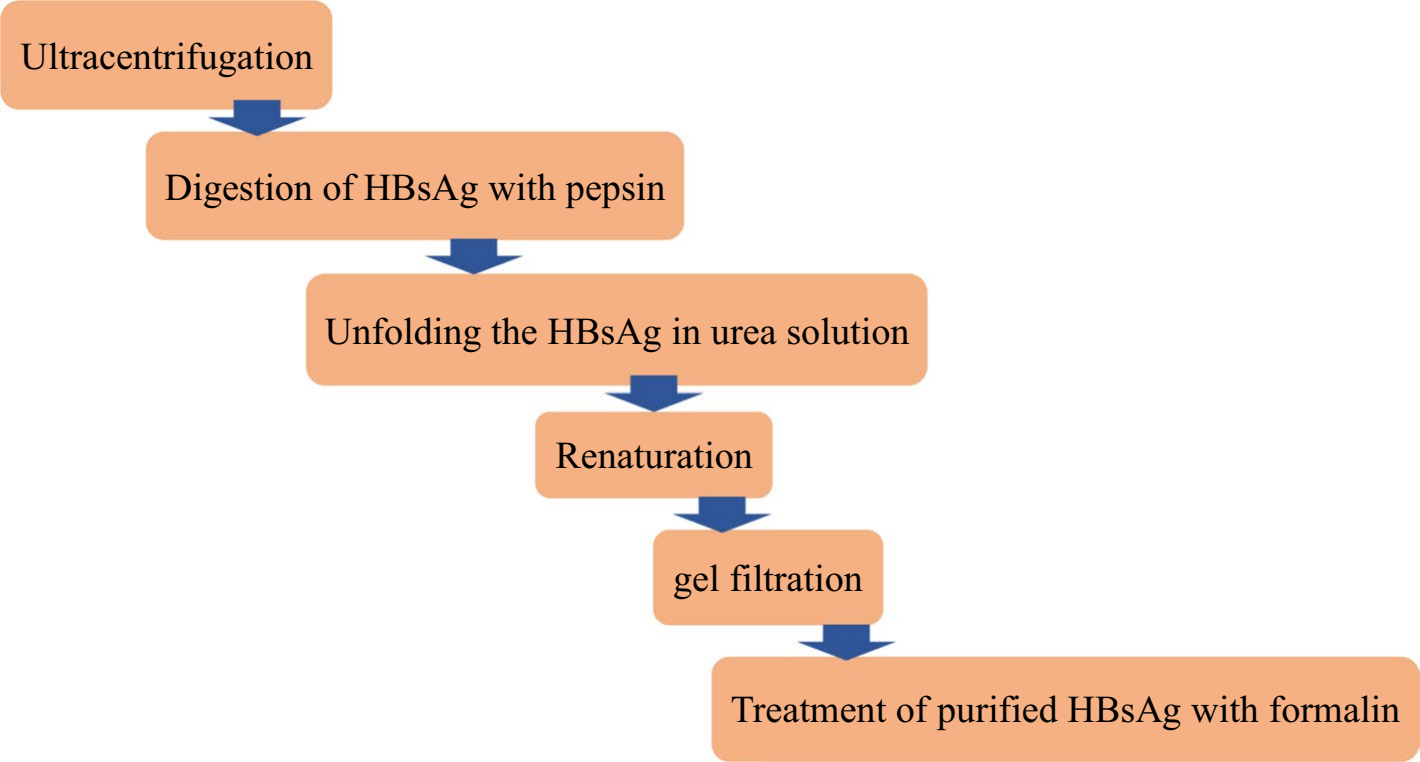
Steps to remove possible pathogens in plasma-derived hepatitis B vaccine preparation

Since 1982, plasma-derived vaccines for HBV were introduced as the first commercially accessible hepatitis B vaccines. They are produced from the inactivated plasma fluid of chronically asymptomatic donors infected with HBV through the collection of hepatitis B surface antigen (HBsAg) subviral particles (SVP) [9, 14]. Following assessment of the success rate of plasma-derived vaccines in millions of people, the first licensed vaccines against HBV were produced for the immunization program in high-risk individuals [15]. The high efficiency and safety of the hepatitis B vaccine in the prevention of asymptomatic infection, acute hepatitis B and chronic HBV carriers have been confirmed by comprehensive studies [12, 16, 17]. Subsequently, the first commercial plasma hepatitis B vaccines, plasma-derived vaccines, were licensed in the USA and in France in 1981 and 1982, respectively [18, 19]. Due to the common “antigenic determinant” presence in isolates of HBV and the presence of HBV in different serotypes, the plasma hepatitis B vaccine was designed to be subtype cross-protective [20]. Considering that the primary source of the plasma-derived vaccine was the plasma of the HBV carriers, there was a possibility for co-infection with various pathogens, including HIV. Therefore, in order to eliminate the contamination of all possible viruses and pathogens, various steps were used in the preparation of the HB first-generation vaccine [21] (Fig. 2).

On the other hand, in North America and Europe, concerns have been raised about the possibility of vaccine plasma contamination with other viruses, including HIV. Based on the findings, these issues were completely unfounded as no contamination with HIV or other pathogens was reported. However, considerations about the possible safety consequences of plasma-derived vaccines eventually stopped the use of first-generation vaccines. Following these challenges, in the mid-1980s, recombinant DNA vaccines were replaced the plasma-derived vaccines.

### Second-generation recombinant HB vaccine

**Fig. 3:**
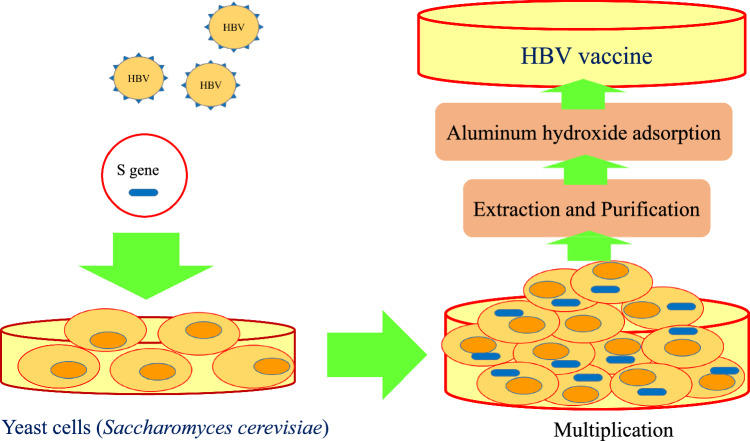
Various steps of second-generation HB vaccines preparation

Second-generation recombinant vaccines were produced, which were progressed as an alternative to plasma-derived vaccines. Large-scale recombinant vaccine production was performed by expressing HBsAg in Saccharomyces cerevisiae and subsequently in mammalian cells [5, 15, 22, 23]. Today, worldwide, the current hepatitis B vaccines in use are entirely of recombinant types [5]. Several steps are required to prepare recombinant vaccines. S gene is isolated from HBV and inserted in yeast cells. Then, during the fermentation process, multitude of HBsAg is produced. The next steps include extraction and purification. Then, to increase the immunogenicity of the HBV vaccine, HBsAg is absorbed on aluminum hydroxide (adjuvant) [5, 24–26] (Fig. 3).

### Third-generation HB vaccines

Despite the safety and immunogenicity of second-generation vaccines, post-translational modifications in a fungal system were still a cause of concern. Therefore, mammalian cells, including mouse-derived cell lines, as well as Chinese hamster ovary (CHO) have been used in the production of third-generation HB vaccines. These cells are able to express and secrete two glycoproteins: small (S), middle (pre-S2/S) or three envelope proteins: large (pre-S1/S2/S) HBsAg. France, Germany, and Korea were among the countries that produced third-generation vaccines by transfecting HBV envelope proteins in mammalian cells [27–29]. Findings have shown that HB vaccines, including pre-S/S viral proteins, are very effective in individuals who are poor responders or non-responders to conventional HB vaccines. Third-generation vaccines can induce antibody response faster than recombinant vaccines produced in yeast [30–32]. Efficacy and immunogenicity of third-generation HBV vaccines are shown in Table 7.

### HB vaccines using adjuvant

In the era of industrial vaccinology, new adjuvants have been used to increase the immune response against the targeted genes. Apart from routine application in current vaccines, adjuvants are intended for use in vaccines for poor responders and non-responders. An HBsAg adjuvanted vaccine with increased immunogenicity using 3-O-desacyl 1–4′monophosphoril lipid A (MPL) and aluminum phosphate has been prepared. Several countries have used these efficient and successful productive vaccines in liver transplant recipients and in hemodialyzed and pre-hemodialyzed patients [33, 34]. Following the third dose administration among non-responders, the seroprotection rate in hepatitis B vaccine (HBsAg/AS04) and Engerix-B vaccines was reported as 98% and 68%, respectively [35]. Appropriate protection/immune response after adjuvanted AS04C hepatitis B vaccination has been reported in autoimmune disease patients [36]. In diabetics, the HBsAg-1018 hepatitis B vaccine has induced a higher seroprotection rate than the HBsAg-Eng hepatitis B vaccine [37]. After three doses of vaccination with HepB-CpG, seroprotection has occurred in all naïve HIV-infected individuals without a history of hepatitis B vaccination [38]. Moreover, seroconversion has been observed to be three times higher in chronic kidney disease individuals who received adjuvanted hepatitis B vaccine compared to non-adjuvanted hepatitis B vaccines [39]. Among hemodialysis adults who were previously non-responders to HB vaccination, a booster dose of the HepB-CpG hepatitis B vaccine produced a greater seroprotection rate than the HepB-AS04 and HepB-Eng vaccines [40]. Recently, a recombinant HB vaccine with CpG (cytosine phosphate guanosine) adjuvant has been approved by FDA. This vaccine induces innate immunity. Individuals receive two doses of the vaccine within a month. This vaccine has been considered for several advantages, including earlier seroprotection, higher seroprotection rate, a shorter schedule and sensible adherence [41]. Some clinical trial studies in non-responders have reported a greater immunogenic response after receiving adjuvanted vaccines [35, 42]. In addition to using adjuvant 3‐O‐desacyl‐4′‐monophosphoryl lipid A (MPL), the greater antigen content used in the Fendrix® vaccine (40‐µg HBsAg) compared to the Engerix‐B® (20 µg) vaccine has induced a greater seroprotection rate [35]. Among 35 non‐responders, seroprotection rate after the two vaccination doses with Engerix‐B® and Heplisav‐B® was demonstrated 66.7% and 88.9%, respectively [42]. Adjuvants, including aluminum adjuvant, are used in vaccine preparation and play a key role in increasing the humoral immune response following vaccination (Table 2). Recently, a novel PF3 nano-adjuvant has been designed to improve the humoral and cellular immune response after hepatitis B vaccination, covering the deficiency of aluminum adjuvant [43].

**Table 2:** Immunogenicity of adjuvanted HB vaccines

Study type	Adjuvanted hepatitis B vaccine	Studied individuals	Age	Gender	Participant (n)	Number of doses	Seroprotection rate	References
Single-blind, randomized/trials	HBsAg/AS04	Healthy non‐responders (majority: healthcare workers)	20–60 years old			0, 1, 6 months	98%	[35]
Controlled phase 2 trial, randomized, Double‐blinded	HBAI20 vaccine	Healthy non‐responders	18 to 59 years	Female: 59 (58.4%), Male: 42 (41.6%)	*n* = 101	0, 1 and 2 months (third doses of vaccination)	80/87 (92.0%)	[44]
Controlled phase 2 trial, randomized, Double‐blinded	HBVaxPro®‐10 µg	Healthy non‐responders	18 to 59 years	Female: 19 (59.4%), Male: 13 (40.6%)	*n* = 32	0, 1 and 2 months (third doses of vaccination)	23/29 (79.3%)	[44]
Open-label study	HBAI20	Non-responders	18 to 59 years	Male: 5 (50%)	*n* = 10	3 vaccinations [0, 1 and 6 months]	90%	[45]
Non-randomized trial, open-label	HBV-AS04	Patients dialysis	67.4 ± 8.4	Male: 149 (60%), Female: 99 (40%)	*n* = 248	four 20-mcg doses * (n = 217): 0,1,2 and 3 months * (n = 31) 0,1,2 and 6 months	202/248 (81.5%)	[46]
Phase III	HB-AS02	Healthy adults	Age of cohort was 29.9 (6.03) years	Females (50.4%)	*n* = 399	Two doses: at 0 and 1 month	* after the first vaccination dose (75.9%) * after two vaccination doses (99.7%)	[47]
Observational study	AS04C	Non-responders			*n* = 195 patients * non-responders: 126 (65%)	four-dose	73.81%	[48]
Observational cohort study	HepB-alum	HIV-infected individuals	Median age: 41 years	Male (78%)	*n* = 59	3-dose	57.6% (seroconversion)	[49]
Observational cohort study	HepB-CpG	HIV-infected individuals	Median age: 41 years	Male (78%)	*n* = 61	2-dose	93.4% (seroconversion)	[49]

#### Efficient and effective conventional hepatitis B immunization

According to the report issued by the World Health Organization (WHO), the hepatitis B vaccine can protect about 100% against HBV infection [1]. Following the valid clinical trial conclusions, vaccination has been considered since the 1980s. Previous studies have shown the effectiveness of the HBV vaccine in preventing perinatal transmission of HBV [50, 51]. Effective HBV vaccination has shown a vital impact in decreasing the incidence rate of HBV-related disorders, the mortality rate of hepatitis B and also the hepatitis B carriership. Since 1984, effective vaccination of infants in Taiwan, as an endemic country, has been very successful. With the initiation of the worldwide vaccination plan, the situation of chronic carrier individuals of the HBV and perinatal transmission of the virus has been dramatically dropped. So, the appropriate amount of antibody levels was detected in about 85% of vaccinated babies at 18 months [52, 53]. A study in China investigated the efficacy of the HBV vaccine in preventing vertical transmission in two groups of infants. One group receiving HBIG plus HBV vaccine and another group receiving only HBV vaccine were evaluated. Interestingly, no vertical transmission occurred after follow-up in any study group [54].

#### Seroprotection failure to conventional HB vaccine

The protective titer in the HBV vaccine is evaluated as a hepatitis B surface antibody (HBsAb) titer > 10 IU/L. However, it is usually unable to provide sufficient protection after exposure to HBV and produces a poor response. Indeed, an adequate and appropriate seroprotective response has the potential to induce HBsAb titer ≥ 100 IU/L (Table 3). Despite the administration of three doses of HBV vaccine, an induction titer < 10 mIU/mL indicates non-responders [55, 56].

**Table 3:** Anti-HBs titers and response to vaccine definition

Anti-HBs titers	Vaccine response status
≥ 10 mIU/mL	Protective
< 10 mIU/mL	Non-responders
10–100 mIU/mL	Low responders
100–999 mIU/mL	Good responders
≥ 1000 mIU/mL	High responders

Today, in spite of available effective HBV vaccines, insufficient immune responses are still observed in certain populations. Therefore, due to the lack of an adequate response, this virus is considered a worldwide cause of concern. For example, people with hepatitis C virus (HCV) infection, human immunodeficiency virus (HIV)-infected individuals, elderly people, smokers, obese people, celiac disease, premature babies, hemodialysis patients, individuals with kidney diseases, chronic liver patients and diabetes mellitus (DM) can be mentioned [57, 58]. A study has shown the association of smoking, obesity and age in individuals with possible HBV vaccination failure [55].

A hepatitis B surface antibody (HBsAb) level of more than 10 IU/L is considered an appropriate and efficient immune response. Hepatitis B virus vaccination aims to induce an adequate host immune response against HBV. Due to the lack of adequate response to the HBV vaccine, especially in people at risk, the rate of infection with the HBV increases as expected. Unfortunately, there is no recommendation for HBV pre-vaccination testing for those at risk. Therefore, in people who do not respond to the vaccine, accurate knowledge of the vaccination history of the virus is not available [58–60]. Babies’ response to the HBV vaccine is completely successful and 100%. On the other hand, they produce HBsAb level > 10 mIU/ml. So, to provide adequate protection in infants, the HBV vaccine without or with hepatitis B immune globulin (HBIG) should be administered approximately 24 h after delivery [61, 62].

Several previous studies have evaluated the response to the HBV vaccine in different populations and separately examined nonresponse among some populations. In one study conducted in vaccinated children, HBsAg was detected at 4.2%, and anti-HBc antibody was identified at 4.8%. Among 165 children who received the HBV vaccine, anti-HBs titer ≥ 10 mIU/ml was reported for 129 (78.2%). Among the 129 vaccinated children, good responders and poor responders to the vaccine were reported in 53 (41.1%) and 76 (58.9%), respectively. Their study showed that although children were vaccinated, a moderate prevalence of HBV infection was due to poor vaccine efficacy in their study population [63]. In low birth weight and preterm infants, the hepatitis B vaccine provides less protection and immunogenicity [64–66]. However, the long-term immunization and protection of infants and children against the HBV vaccine seems to be challenging and controversial.

In a study conducted on dialysis patients receiving the HBV vaccine, the rate of HBV vaccine non-responders was reported as 52.3%. Considerably, HBV vaccine non-responders were older than HBV vaccine responders [67]. Hepatitis B virus (HBV) infection is a life-threatening factor in hemodialysis children with chronic kidney disease. One study evaluated HBV vaccine response rates in hemodialysis children, and 33.8% of these children were HCV antibody positive. The results of their study reported 30% and 70% of children higher than 100 IU/mL (great response level) and ≤ 100 IU/mL (hypo-/non-responders), respectively [68].

Unfortunately, non-responders are significant reservoirs for HBV transmission and are considered HBV carriers. Therefore, despite the availability of a successful HBV vaccine, hepatitis B is still considered a critical problem in various populations.

One of the most important factors influencing the response to the HBV vaccine is the age of individuals. It seems that with increasing age and gradually, the level of antibody produced in response to the HBV vaccine decreases significantly. On the other hand, typically, poor responses are observed in approximately 10% of people [69]. It seems that seroprotection failure and poor responses to individuals to the HBV vaccine can significantly affect the effectiveness of the vaccine. Overall, the possible mechanisms of inadequate HBV vaccine response in different populations are quite different depending on multiple factors and are not comprehensively understood. Therefore, more studies and reviews are needed (Table 4).

**Table 4:** Response status to conventional HB vaccine

Studied subjects	Vaccinated subjects	Age	Gender	Vaccine doses	Non-responders anti-HBs titers < 10 mIU/mL	Low-responders anti-HBs titers 10–100 mIU/ml	Good responders > 100 mIU/mL	References
Students	*n* = 1704		Females: 1033 (60.6%)	Three doses	270 (15.8%)	* 10–400 IU/L 987 (57.9%)	* > 400 IU/L 447 (26.3%)	[70]
Children	*n* = 1814		Male: 1006 Female: 808	Three doses (5 μg) 0, 1 and 6	3.1%	28.9%		[71]
Children	*n* = 3752	6 years	Male: 2456 (65.4%) Female: 1302 (34.6%)	Three doses	723 (19.3%)	1939 (51.6%)	1096 (29.2%)	[72]
Healthcare workers	*n* = 200	19 to 52 years old		Three doses	7 (3.5%)	22 (11.0%)	171 (85.5%)	[73]
Healthcare Workers, Medical students	*n* = 340	18 to 60 years	Females: 204 (60%) Males: 136 (40%)	Three doses	n = 40	* > 10 mIU/ml n = 300		[74]
Healthcare workers	*n* = 166			Three doses 0, 1 and 6 months	50 (30%)	18 (10.8%)	98 (59.2%)	[75]
Medical Staff	*n* = 239	20–55 years	Male: 43 (18%) Female: 196 (82%)	Three doses	14 (5.9%)	37 (15.5%)	188 (78.6%)	[76]
Healthcare workers	*n* = 652	Majority: < 25–39 years old	Females: 271 (41%) Males: 381(59%)	Three doses 0, 1 and 6 months	< 25 years: 23 (9%) 25–34 years: 41 (13.0%) 35–49 years: 19 (26%) ≥ 50 years: 7 (63%)	*Normal responders: < 25 years: 234 (91%) 25–34 years: 270 (87%) 35–49 years: 54 (74%) ≥ 50 years: 4 (36%)		[77]
Healthcare workers	*n* = 151 HBV-vaccinated subjects = 129	20–59 years	Males: 24 (15.9%) Females: 127 (84.1%)		26 (17.2%)	*anti-HBs titer > 10 103 (68.2%)		[78]
Medical staff	*n* = 49				20.4%	34.7%	44.9%	[79]
Diabetic children	*n* = 110	2–23 years	Male: 75 Female: 35	Three doses	46 (41.8%)			[80]
Children	*n* = 427	6 year old	Female: 223 Male: 204	Three doses	105 (24.6%)	181 (42.3%)	141 (33.1%)	[81]

#### Occult hepatitis B infection (OBI) following HB vaccination

**Fig. 4:**
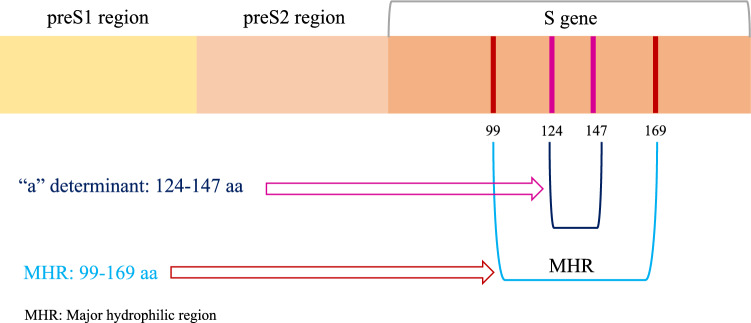
Occult hepatitis B virus infection (OBI) associated with “a” determinant mutation

Although successful universal immunization of newborns is carried out, surface antigen vaccine-escape mutation and OBI infection are considered to be interfering factors in the eradication of HBV infection [82]. The precise mechanism of OBI is not fully understood. Nevertheless, one of the identified mechanisms of OBI is “a” determinant mutation of HBsAg [83]. Creating a mutation in “a” determinant region due to conformational changes of HBsAg leads to a decrease in HBV diagnostic and, subsequently, serological detection failure [84]. The G145R mutation has been distinguished as the most common and well-known HBsAg mutation, which plays an important role in vaccine escape [84]. A region of surface protein (HBsAg) including major hydrophilic region (MHR) domain consists of 99–169 aa. In addition, “a” determinant is located in the region between 124 and 147 aa. On the other hand, “a” determinant mutations are associated with vaccine-escape mutants, and subsequently, OBI occurs (Fig. 4) [85].

In the 1970s, despite the negative results of serum markers, including HBsAg negative, the possibility of another profile of HBV infection was reported [86, 87]. After the development of advanced molecular techniques with great sensitivity, silent or OBI following HBV infection has been characterized [88, 89]. In fact, in this phase of chronic HBV infection, the viral genome is present in the episomal form of covalently closed circular DNA (cccDNA) with low replication conditions, which challenges the detection of HBV DNA in plasma or serum, and if detectable, a low viral level indicates approximately < 200 IU/mL. Therefore, several factors affect the detection of HBV DNA in plasma/serum (Table 5). Among these multiple factors, the sensitivity of the performed technique, specimen volume, the studied subjects and the status of collection of the analyzed blood specimens can be considered [90–95]. Occult hepatitis B infection (OBI) is distinguished by a negative HBsAg status, the detection of very low levels of HBV-DNA replication in the liver and the absence or presence of HBV DNA in the blood [95].

**Table 5:** Clinical implications in occult hepatitis B infection (OBI) phase of HBV infection

Occult hepatitis B infection (OBI)							
Seropositive OBI				Seronegative OBI			
HBV DNA in serum	HBsAg	Anti-HBc IgG	Anti-hepatitis B surface (HBs) IgG	HBV DNA in serum/liver tissue	HBsAg	Anti-HBc IgG	Anti-hepatitis B surface (HBs) IgG
Detectable	Negative	Positive	Positive/Negative	Detectable	Negative	Negative	Negative

Although in the diagnosis of OBI, viral load < 200 IU/mL is determined for patients, nevertheless, in ˃ 90% of patients diagnosed with OBI, the level of viral load in the serum has been detected at approximately 20 IU/mL [96]. A study conducted in China reported significantly higher rates of maternal levels of viral loads greater than 100 IU/mL among OBI-positive babies compared to infants with negative results for OBI [97]. In adults, roughly 1–3% of OBI have been identified following HB vaccination [98, 99]. In a cohort study, among vaccinated subjects, the prevalence of OBI in anti-HBc-positive individuals 16/334 (4.8%) was greater than that of anti-HBc-negative individuals: 0/392 (0%) [100]. Another study on HB-vaccinated blood donors demonstrated three primary OBIs and 17 OBIs [101]. The prevalence of OBI in children is not fully understood, but it is potentially possible. In childhood and infancy, HBV infection is easily transmitted. Babies infected with HBV account for 25–30% of chronic carriers until the end of life. Therefore, progression to liver cirrhosis and liver cancer occurs at a higher rate in these children compared to being HBV-infected at an older age. Several possible factors, including high viral load levels in the mother, hyporesponse/nonresponse to the HB vaccine, decreased titers for anti-HBs protection and the development of mutations in the S region associated with vaccine escape, could play a role in OBI among HB-vaccinated children [97]. Several studies have reported the prevalence of OBI in HBV-vaccinated individuals in different populations (Table 6).

**Table 6:** Prevalence of occult hepatitis B infection (OBI) among vaccinated individuals

Studied population	*N*	OBI Number N (%)	Factors involved	Country	References
HBV vaccinated children	*N* = 46	5 (10.9)	C139S vaccine-escape mutant, Variation and deletion in pre-S region	Taiwan	[102]
Anti-HBs-positive young adults	*N* = 2919	124 (4.2)	Mutations at the “a” epitope/outside of the “a” epitope	China	[99]
Children with HBsAg-positive mothers	*N* = 75	21 [28]	13 (62%): at least one mutation, 10: G145R mutations	Iran	[103]
Outpatients	*N* = 282	1		Tanzania	[104]
Newborns with HBsAg positive mothers	*N* = 100	2 [2]		African (French island in the Mozambican canal)	[105]
School healthy children	*N* = 229	Five cases	Amino acid mutation in S region/Pre-S region	Indonesia	[106]
Babies with HBsAg-positive mothers	*N* = 213	89 [42]		India	[107]
Immunized children	*N* = 327	10 (3.1)		China	[108]
HB vaccinated babies with HBsAg-positive mothers	*N* = 183	9 (4.92)	Maternal viral loads > 100 IU/mL, Four pre-S/S sequences of C/D genotype: S143L escape mutation	China	[97]

#### Individuals at risk of HBV infection following HB vaccine Failure

A study demonstrates the importance of HB vaccination in healthcare workers (HCWs). Most HCWs are unvaccinated. Anti-HBs titers decrease gradually over time in HB vaccinated individuals. In a significant population of HCWs who have received full doses of vaccination, as well as partially vaccinated individuals, protective titers against HBV infection are inadequate. The study emphasizes the importance of universal screening for anti-HBs titer, HBs antigen measurement and administration of booster dose of HB vaccine in HCWs [75]. Given the ability of HBV to cause chronic and sub-clinical infection, the ability to be transmitted through body fluids and blood in non-immune individuals, as well as vaccination failures, especially in healthcare systems, transmission may occur inadvertently. Revaccination has been considered an appropriate solution in non-responders/low-responders in developed countries. Subsequently, seroconversion has been achieved in most low-responders. However, after revaccinations, approximately half of non-responders do not increase HBs antibody levels. A study reported that a patient developed acute hepatitis B infection despite receiving five doses of HB vaccine. The primary response to HB vaccination in this patient was moderate, with a high level of anti-HBs > 1000 IU/l being reported after booster HB vaccination (recombinant DNA vaccine). However, this patient showed HBV infection and developed acute hepatitis 14 years after this booster recombinant DNA vaccination. Their study described hepatitis B vaccination failure even after a high level of anti-HBs was raised following recombinant DNA hepatitis B vaccination [109].

#### Reducing hepatocellular carcinoma (HCC) rates following HB vaccination

One of the most important malignancies worldwide is liver cancer, which is known as the third leading cause of mortality rate among various cancers [110]. Hepatocellular carcinoma (HCC) is one of the most challenging and important public health problems. Approximately 90% of primary liver cancer cases are attributed to HCC. One of the obstacles to the effectiveness of treatment is the late diagnosis of HCC in the advanced phase of the disease [111]. The prevalence rate of HBV infection, as well as the incidence of HCC, has been significantly reduced following universal HB vaccination [112, 113]. Fortunately, effective HB vaccination can prevent HBV and the progression of the disease to HCC.

#### Improved response through hepatitis B third-generation vaccines

Recent studies have demonstrated that a tri-antigenic hepatitis B vaccine (S, pre-S1, and pre-S2 antigens) induced a more robust immune response compared to a mono-antigenic hepatitis B vaccine (S antigen) [114, 115] (Table 7).

**Table 7:** Efficacy and immunogenicity of third-generation HBV vaccines

Type of study	Study subjects	Viral antigens/Proteins	Number of studied subjects	Age	Gender	Number of doses	Seroprotection rate (SPR)	References
Phase 3 trial, double-blind, randomized,	Adults	S, pre-S1, and pre-S2 antigens	*n* = 718	≥ 18 years		Third vaccination (on days 0, 28, and 168)	656 (91·4%)	[114]
Phase 3 trial, double-blind, randomized,	Adults	S, pre-S1, and pre-S2 antigens	*n* = 625	≥ 45 years		Third vaccination (on days 0, 28, and 168)	559 (89·4%)	[114]
Randomized Clinical Trial, Phase 3	Healthy adults	3-antigen (3A)-HBV	*n* = 1753	18 to 45 years		Third Injection	1740 (99.3%)	[115]
Clinical Trial, Randomized, Phase 3	Healthy volunteer	3-Antigen (Pre-S1/Pre-S2/S)	*n* = 50	18–45 years	Male [18] Female [32]	3-dose regimens of 3AV (10 μg)	100%	[116]
Phase IV study	Healthy young adults	3-Antigen (Pre-S1/Pre-S2/S)	*n* = 91	20–40 years	Male [74] Female [17]	Three IM doses of 10 μg (at 0, 1 and 6 months)	Seroprotective levels: 100% * at month 7: 97.6% (n = 81), high responders (≥ 100 mIU/mL)	[117]
Prospective cohort	HIV-infected adults	Sci-B-Vac™: mimics 3-Antigen (Pre-S1/Pre-S2/S)	*n* = 31			10 µg/three dose/intramuscularly/0, 1 and 6 months	84%	[118]

#### The role of various host genetic factors

Recently, the critical role of genetic factors involved in the non-responders to the HBV vaccine has been identified. The most important factors include single nucleotide polymorphisms (SNPs) in toll-like receptors (TLRs), cytokine receptors/cytokine, chemokine, and human leukocyte antigen (HLA) haplotypes [119].

### Single nucleotide polymorphisms (SNPs) in chemokine and cytokine genes

Single nucleotide polymorphisms (SNPs) occurring in the IL-4 and IL-2 loci associated with insertion/deletion (indels) variants in the IL12B gene are closely related to HBV vaccine response status [120]. Furthermore, the prominent role of chemokines and cytokines in HBV vaccine response has been identified. For example, one SNP (rs355687) in CXCL13 and three SNPs (rs3922, rs497916, rs676925) in CXCR5 have been detected, which have shown a close relationship with the HBV vaccine response [121]. Also, a significant relationship between SNPs and hepatitis B vaccine host response was reported. Interestingly, a potential genetic relation was identified between the major histocompatibility complex (MHC) locus located on the chromosome 6 (rs5000563) and hepatitis B vaccine response. On the other hand, based on possible predictions, other SNPs in this region are able to change the sequence of proteins [122].

### Human leukocyte antigen (HLA)

Several studies have reported the relation of HB vaccine response with genetic variation identified at the HLA locus [120, 122–124]. A low rate of HLA-CW6 and a high rate of HLA-A24 and HLA-A11 have been demonstrated to be related to non-responders HB vaccine [125]. Non-response to HB vaccine has been reported in both healthy individuals and patients with celiac disease associated with DQ2, DR3 and HLA-B8 haplotypes [126]. It has been reported that although HLA-B13 is associated with adequate response to vaccine, however, HLA-DRB1*0401X0201, DRB1*11/13, DRB1*0401X and DRB1*04X haplotypes are associated with no-response [127]. Furthermore, the association of poor response to the HB vaccine with some HLA alleles, such as DRB1*08(-), DRB1*07 and B62, has been identified [128]. Associations between poor vaccine responses and DRB1*04, DRB1*07, DRB1 *03 (DRB1*0301), DRB1*1302 and DQB1*02 HLA alleles have also been identified [129]. Recently, one cohort study evaluates the relationship between response vaccine antigens and HLA in Bangladeshi children. Association of greater HBV antibody response with DPB1*04:01 was reported. Hepatitis B surface antigens (HBsAg) epitopes bind with higher affinity to DPB1*04:01 dimers. Most probably, the evolutionary pressure created in the “a” determinant region leads to the HBV vaccine-escape mutants [130]. Various main factors related to poor/low response to HBV vaccination are demonstrated in Table 8.

**Table 8:** Various main factors associated with poor/low response to HBV vaccination

Main factors	References
Inappropriate storage condition during transport of vaccine	[131]
Smoking	[55, 69, 132]
HIV infection	[133]
Obesity	[55, 69, 79, 132]
Diabetes	[76, 80, 134]
Insulin-resistance	[79]
HLA haplotypes	[130, 135]
Celiac disease	[136–140]
Gender (males)	[69, 77, 79]
Age	[55, 69, 73, 76, 77, 79, 132]
HCV	[141–143]
Hemodialysis patients	[144]
Peritoneal dialysis patients	[145]
Chronic kidney disease	[146]
Genetic factors	[147]
Body mass index (BMI)	[132]
Low birth weight	[71]
Immune cells	[148]
Immunosuppressive drugs	[76]

#### Hepatitis B vaccine-escape mutants

HBV surface antigen (HBsAg) is known as the main envelope protein of the virus. On the other hand, HBsAg contains the main epitopes as well as the important regions involved in the binding of HBV to hepatocytes, which neutralizing antibodies recognize the HBsAg [149]. Protection against HBV infection and immunity undergo changes following mutations in the “a” determinant. These changes can induce HBV vaccine evasion [150]. In 1988, these mutations’ vital impact and importance were reported through babies with HBsAg-positive mothers for the first time. In these infants, infection was identified despite administering the HBV vaccine and HBIG. This mutation causes a change in “a” determinant. As a result, it is impossible to identify the virus through neutralizing antibodies produced after vaccination, and following this significant alteration, infection occurs [150]. The mechanism of mutation in the S gene is not fully understood; however, some hypotheses have suggested the occurrence of these spontaneous mutations in the S gene due to the interference of the host’s immune system [151, 152]. In Taiwan, out of 12 children who received the HBV vaccine, 8 were identified as detectable HBV DNA-positive in serum. One of them demonstrated “a” determinant mutation [153]. However, other HBV vaccine-escape mutations such as M133L, P120S/E, T116N, Q129H/R, I/T126A/N/I/S, D144A/E, D144A/H, P142S, G145R/A, K141E, A128V, G130N and M133L/T have been identified to be associated with the “a” determinant. Still, despite widespread vaccination, the G145R as predominant circulating escape mutant has been reported [149, 154–157]. One case report study demonstrated that an infant developed an occult HBV infection (OBI) after a liver-transplantation despite receiving the HBV vaccine. Specific vaccine-escape mutations have been shown to play a critical role in the transmission of OBI [158]. A case report study has shown that despite receiving the vaccine, a person progressed to an acute hepatitis B infection. Q129H vaccine-escape mutation in the “a” determinant with the mechanism of altering HBsAg antigenicity, and also critical and effective change in the attachment of anti-HBs to HBsAg, has been involved in vaccine evasion [159]. Recently, HBV-specific vaccine-escape mutations have been reported in Bangladesh [160]. In addition, in the Netherlands, HBV vaccine-escape mutations in the HBsAg due to genetic changes have been identified [161]. These mutations have the ability to prevent the recognition of HBsAg by antibodies and can also control the secretion of HBsAg [162]. Vaccine-escape mutations related to hepatitis B surface antigen “a” determinant region/major hydrophilic region are demonstrated in Table 9.

**Table 9:** Vaccine-escape mutations associated with hepatitis B surface antigen “a” determinant region/major hydrophilic region

Amino acid substitutions/Position of codon	References
G145R/A	[103, 150, 153, 157, 158, 161, 163–176]
T116N	[177]
M133L	[178, 179]
P120S/E	[167, 168, 180]
D144A/E	[161, 169, 179, 180]
I/T126A/N/I/S	[169, 180]
K141E	[166]
Q129H/R	[159, 161, 178, 179]
P142S	[169, 170]
N146S	[153]
T131I	[171]
Ile/Thr-126-Asn/Ser	[173]
I126S/N	[157]
T126A	[157]
128 V	[160]
Y100C	[161]
L109I	[161]
T118R	[161]
P120T/S	[161]
T126I/S/A	[161]
P127T/L	[161]
T131S/I	[161]
M133I	[161]
F/Y134N/L	[161]
T140I	[161]
S143L	[161]
A168V	[161]
T126I	[158]
P120T	[158]
P142S	[158]
M133I	[176]

#### Suggested solutions

In non-responder individuals who do not develop adequate seroprotection after three doses of conventional HB vaccination, 1 to 3 additional doses of HB vaccine are usually recommended [42]. In high-risk individuals with a history of HB vaccination and reduced titers (anti-HBs < 10 mIU/ml), a booster dose is recommended for a protective effect [181]. Receiving a fourth dose of HB vaccine has been reported as an efficient strategy in HCV-infected individuals [142]. Although some studies have reported a poor response to HBV vaccination despite treatment, especially in patients with chronic HCV [182, 183], HBV revaccination after treatment of HCV infection is recommended for non-responders [184]. On the other hand, despite receiving a booster dose of HBV vaccine, celiac patients are still considered to be poor responders to the vaccine [140]. Therefore, according to the controversial findings, it seems that achieving a hepatitis B vaccine with long‐term protection is still challenging. In addition, after receiving the 3-antigenic hepatitis B vaccine, more robust and faster seroprotection is induced, which can provide earlier seroprotection than the mono-antigenic vaccine. The results obtained from recent studies highlight the clinical importance of using third-generation vaccines, especially in non-responders and susceptible high-risk individuals at risk of HBV infection [114, 115]. Also, some studies have new suggestions. One case report study demonstrated that protection was induced by intradermal HBV vaccination in HBV vaccine non-responder HIV-infected patients [185]. Among general persons and hemodialysis who were non-responders to intramuscular HB vaccination, intradermal HB vaccination has produced an efficient response [186]. Recently, immunogenicity and safety of therapeutic HBV vaccine have been demonstrated in both chronic HBV patients and healthy individuals [187]. According to the best of our knowledge, in addition to receiving booster doses, the hepatitis B third-generation vaccines and new adjuvanted recombinant HB vaccines are targeted and highly effective alternatives to conventional HB vaccines, and universal use of these new comprehensive approaches is recommended.

## Conclusion

Despite the occurrence of HB vaccine-escape mutations and the decrease in HBsAb titer over time, the best and most important strategy against this blood-borne virus is still universal vaccination. In contrast, depending on the development of HBsAg-specific mutations and, as a result, the impairment of HBsAg recognition by neutralizing antibodies and the genetic factors of the host involved in the response to the vaccine, the efficiency of HBV vaccination is affected. Therefore, despite the highly efficient HBV vaccine, producing a vaccine with long-term protection in all targeted Individuals is still very challenging. In addition, monitoring and following the HBsAb titer continuously is an appropriate solution for high-risk individuals and healthcare workers, especially in endemic regions. If the HBsAb level decreases in these individuals, it is suggested that a booster dose be prescribed if needed. The HB vaccination strategy remains valuable despite viral and host factors involved in HB vaccine failure. Fortunately, not only receiving booster doses in high-risk groups is an appropriate approach, but also the use of new adjuvant vaccines and third-generation HB vaccines is recommended as the best alternatives to conventional HB vaccines, particularly in no/poor responders. However, this issue should be followed quite seriously.

## Data Availability

No datasets were generated or analyzed during the current study.
